# Supplementary material for: HMG-CoA reductase inhibitors and COVID-19 mortality in Stockholm, Sweden: A registry-based cohort study
Source: PLoS Med. 2021 Oct 14;18(10):e1003820. doi: 10.1371/journal.pmed.1003820 (PMC8516243; doi:10.1371/journal.pmed.1003820)
Supplement: S1 Table — (DOCX) [file pmed.1003820.s002.docx]

| **S1 Table. Covariate definition according to ICD-10/ATC-code and period of data collection.** | | |
| --- | --- | --- |
| **Covariate** | **ICD-10 /ATC-code** | **Period** |
| Cancer | C00-C99 | 2015-01-01 - 2020-02-29 |
| Diabetes type I | E10 | 2015-01-01 - 2020-02-29 |
| Diabetes type II | E11 | 2015-01-01 - 2020-02-29 |
| Obesity | E66 | 2015-01-01 - 2020-02-29 |
| Dyslipidaemia | E78 | 2015-01-01 - 2020-02-29 |
| Dementia (inc. Alzheimer's disease) | F00-F03, G30 | 2015-01-01 - 2020-02-29 |
| Other neurological conditions | G10-G14, G20-G26, G31, G32, G35-G37, G70-G73, G80-G83 | 2015-01-01 - 2020-02-29 |
| Hypertension | I10-I15 | 2015-01-01 - 2020-02-29 |
| Heart failure | I42, I43, I50 | 2015-01-01 - 2020-02-29 |
| Ischemic Heart Disease | I20-I25 | 2015-01-01 - 2020-02-29 |
| Atrial fibrillation | I48 | 2015-01-01 - 2020-02-29 |
| Cerebrovascular disorders | I60-I69 | 2015-01-01 - 2020-02-29 |
| Vascular disease | I70-I79 | 2015-01-01 - 2020-02-29 |
| Chronic lower respiratory disease | J40-J44, J47 | 2015-01-01 - 2020-02-29 |
| Chronic kidney disease | N18 | 2015-01-01 - 2020-02-29 |
| Peripheral vascular disease | I73.9 | 2015-01-01 - 2020-02-29 |
| Stroke and TIA | I63-I69, G459 | 2015-01-01 - 2020-02-29 |
| Renal failure stage 3-5 | N18.3, N18.4, N18.5 | 2015-01-01 - 2020-02-29 |
| Hypercholesterolemia or mixed hyperlipidaemia | E78.0, E78.2, E78.5 | 2015-01-01 - 2020-02-29 |
| ACE inhibitors | C09A | 2019-03-01 - 2020-02-29 |
| Angiotensin receptor blockers | C09C | 2019-03-01 - 2020-02-29 |
| Anticoagulants | B01A | 2019-03-01 - 2020-02-29 |
