# Supplementary material for: HMG-CoA reductase inhibitors and COVID-19 mortality in Stockholm, Sweden: A registry-based cohort study
Source: PLoS Med. 2021 Oct 14;18(10):e1003820. doi: 10.1371/journal.pmed.1003820 (PMC8516243; doi:10.1371/journal.pmed.1003820)
Supplement: S2 Table — (DOCX) [file pmed.1003820.s003.docx]

| **S2 Table. Hazard ratios of death from COVID-19 and death from other causes in relation to dispensations of statins overall, and the hazard ratios within strata of age, sex and within COVID-19 risk groups, estimated using the Fine-Gray subdistribution hazard model.** | | | | | | | | |
| --- | --- | --- | --- | --- | --- | --- | --- | --- |
|  | **Number Covid-19 deaths** | | **Number other causes of deaths** | | **Subdistribution hazard model**  **HR (95% CI)** | | | |
|  | Statin users | Non-statin users | Statin users | Non-statin users | Covid-19 death | | Other causes of death | |
|  |  |  |  |  | Unadjusted | Fully adjusted | Unadjusted | Fully adjusted |
| ***Overall*** | 765 | 1 780 | 2 350 | 6 149 | 2.01 (1.85-2.19) | 0.88 (0.80-0.98) | 1.80 (1.71-1.88) | 0.78 (0.73-0.82) |
|  |  |  |  |  |  |  |  |  |
| **Age** |  |  |  |  |  |  |  |  |
| *45-69* | 85 | 215 | 297 | 1 175 | 3.18 (2.48-4.09) | 0.79 (0.55-1.13) | 2.03 (1.79-2.31) | 0.63 (0.53-0.75) |
| *70-79* | 230 | 340 | 785 | 1 391 | 1.34 (1.14-1.59) | 0.90 (0.73-1.11) | 1.12 (1.03-1.22) | 0.71 (0.63-0.79) |
| *80+* | 450 | 1 225 | 1 268 | 3 583 | 0.71 (0.64-0.79) | 0.84 (0.74-0.96) | 0.68 (0.64-0,72) | 0.80 (0.74-0.87) |
| **Sex** |  |  |  |  |  |  |  |  |
| *Male* | 502 | 861 | 1 428 | 2 747 | 2.26 (2.02-2.52) | 0.90 (0.79-1.03) | 2.02 (1.89-2.15) | 0.75 (0.69-0.81) |
| *Female* | 263 | 919 | 922 | 3 402 | 1.65 (1.43-1.89) | 0.86 (0.73-1.00) | 1.56 (1.45-1.68) | 0.81 (0.74-0.88) |
| **Covid-19 risk group** |  |  |  |  |  |  |  |  |
| *No known risk factor* | 404 | 1 384 | 1 256 | 4 936 | 1.79 (1.61-2.00) | 0.90 (0.80-1.02) | 1.57 (1.47-1.67) | 0.80 (0.75-0.86) |
| *Ischemic Heart Disease* | 307 | 261 | 908 | 737 | 0.37 (0.32-0.44) | 0.75 (0.61-0.91) | 0.39 (0.35-0.43) | 0.67 (0.60-0.76) |
| *Hypertension* | 652 | 1 127 | 1 983 | 3 602 | 0.90 (0.82-0.99) | 0.85 (0.75-0.95) | 0.86 (0.81-0.90) | 0.75 (0.70-0.80) |
| *Diabetes type II* | 369 | 336 | 919 | 934 | 0.75 (0.65-0.87) | 0.86 (0.73-1.03) | 0.67 (0.61-0.73) | 0.69 (0.62-0.77) |
| *Chr lower resp tract dis* | 131 | 229 | 470 | 888 | 1.11 (0.90-1.38) | 0.86 (0.66-1.12) | 1.03 (0.92-1.15) | 0.80 (0.70-0.92) |
| *Stroke and TIA* | 31 | 70 | 79 | 256 | 2.10 (1.38-3.20) | 1.22 (0.70-2.12) | 1.47 (1.14-1.89) | 0.60 (0.44-0.83) |
| *Chr kidney failure st 3-5* | 9 | 44 | 41 | 114 | 0.97 (0.47-1.99) | 0.59 (0.25-1.40) | 1.71 (1.20-2.44) | 0.62 (0.38-1.02) |
| *Heart failure* | 236 | 412 | 793 | 1 367 | 0.60 (0.51-0.71) | 0.78 (0.64-0.95) | 0.60 (0.55-0.66) | 0.74 (0.66-0.82) |
