# Supplementary material for: HMG-CoA reductase inhibitors and COVID-19 mortality in Stockholm, Sweden: A registry-based cohort study
Source: PLoS Med. 2021 Oct 14;18(10):e1003820. doi: 10.1371/journal.pmed.1003820 (PMC8516243; doi:10.1371/journal.pmed.1003820)
Supplement: S1 Text — (DOCX) [file pmed.1003820.s004.docx]

## Emulated target trial

We base the design of our investigation on the so-called emulated target trial framework - a framework utilized to minimize self-inflicted injuries when estimating causal effects using observational data [1]. Specifically, we attempt to emulate (replicate) a hypothetical trial of the primary prevention effect of statin on COVID-19 mortality. However, in contrast to a hypothetical trial, we chose to include prevalent statin users. This greatly enhances the eligible population but may lead to the inclusion of a selected population of therapy sustainers (and depletion of susceptibles).

To alleviate such bias, we repeat our analysis excluding prevalent users. Specifically, we emulated a target trial by excluding all individuals who received one or more prescriptions for statins during the year before the start of the study period (1 March 2018 – 28 February 2019). This means that only new users (initiators), not prevalent users, were included in the analysis. The analysis did not take into account possible discontinuation of treatment during the study period, meaning that the emulated trial was done according to the intention-to-treat principle.

We identified 22 943 (2.9%) new statin users (i.e. who had not collected any statin prescriptions for a year before the study period) meeting the inclusion criteria. The negative association between statin treatment and COVID-19 mortality remained after the exclusion of prevalent users, but was not statistically significant (adjusted HR, 0.78; 95% CI, 0.59-1.05). Restricting the emulated trial to those with probable indication for statin treatment did not alter our findings (adjusted HR, 0.73; 95% CI, 0.48-1.10). Specifically, in accordance with standard practice under the emulated target trial framework we exclude those without known indication for statin treatment (hypercholesterolemia, ischemic heart disease, peripheral vascular disease, stroke and TIA, and renal failure stage 3-5) and repeated our analyses, adjusting for covariates which did not define the strata.

## COVID-19 as main underlying cause of death

We identified 2 377 individuals who had COVID-19 registered as the main underlying cause of death, of which 716 were statin users and 1 661 were not statin users (HR, 2.02; 95% CI, 1.85-2.21). When adjusting for confounders, there was a negative association between statins and COVID-19 as main underlying cause (HR, 0.88; 95% CI, 0.79-0.97), similar to that of our main analysis.

## Analysis of all-cause mortality as a positive outcome

To further triangulate our findings, we performed an analysis of all-cause mortality, excluding COVID-19 deaths, as the outcome (“positive outcome analysis”) [2]. As an external validation of our positive outcome estimate, we compared our finding to that of a meta-analysis of randomized controlled trials examining statins for primary prevention in high-risk populations (RR, 0.91; 95% CI, 0.83-1.01) [3]. In the absence of bias, we would expect our positive outcome estimate to be very similar to that of the selected meta-analysis. An estimated positive (or null) association between statins and all-cause mortality would imply the possibility that our studied cohort suffers from some intrinsic bias (possibly selection bias), which may make our COVID-19 analysis biased, under an assumption of so-called biased equivalence (i.e., any bias affecting the positive outcome will similarly affect the outcome of interest) [2].

We identified 8 499 deaths during the study-period which were not attributable to COVID-19 (no registered diagnosis), of these 2 350 were statin users and 6 149 were not statin users. After adjusting for confounders there was an inverse association between statin treatment and all-cause mortality (HR, 0.77; 95% CI, 0.73-0.82), consistent with that of the meta-analysis of randomized controlled trials examining statins for primary prevention in high-risk populations, albeit with a greater magnitude.

## References

1. Hernan MA, Robins JM. Using Big Data to Emulate a Target Trial When a Randomized Trial Is Not Available. Am J Epidemiol. 2016;183(8):758-64.

2. Tchetgen Tchetgen E. The control outcome calibration approach for causal inference with unobserved confounding. Am J Epidemiol. 2014;179(5):633-40.

3. Ray KK, Seshasai SR, Erqou S, Sever P, Jukema JW, Ford I, et al. Statins and all-cause mortality in high-risk primary prevention: a meta-analysis of 11 randomized controlled trials involving 65,229 participants. Arch Intern Med. 2010;170(12):1024-31.
