# Supplementary material for: HMG-CoA reductase inhibitors and COVID-19 mortality in Stockholm, Sweden: A registry-based cohort study
Source: PLoS Med. 2021 Oct 14;18(10):e1003820. doi: 10.1371/journal.pmed.1003820 (PMC8516243; doi:10.1371/journal.pmed.1003820)
Supplement: Attachment — Submitted filename: Rebuttal - Point by Point.docx [file pmed.1003820.s005.docx]

Requests from the editors:

**Please update the title of the manuscript to match the style used by PLOS Medicine. “Prescriptions for HMG-CoA reductase inhibitors and COVID-19 mortality in Stockholm, Sweden: a registry-based cohort study”, or similar, would be suitable.**

**Answer:** We have now changed the title of the manuscript to ensure compatibility with the style of PLOS Medicine. We choose to leave out “prescription” since our exposure is drug dispensation, and not only a prescription per se. We hope this accommodates the editors. The title now reads as:

HMG-CoA reductase inhibitors and COVID-19 mortality in Stockholm, Sweden: a registry-based cohort study

**Please confirm whether ‘Swedish secrecy law’ (in your response to the data availability question in the submission form) should be updated to ‘Swedish privacy law’.**

**Answer:** We confirm that we are invariant to such an update. It is a general English translation of a group of legal documents concerning patient data safety.

**Please structure the ‘Abstract’ using three sections: ‘Background’, ‘Methods and Findings’, and ‘Conclusions’. The ‘Objectives’ section in the current abstract can be rephrased as the final line of the ‘Background’ (“The objective of this study was to determine the relationship…” etc.). The ‘Methods’ and ‘Results’ sections can be combined, and the final sentence of the ‘Methods and Findings’ section ought to summarise 2-3 of the study’s main limitations (please begin this sentence “Limitations of this study include…”).**

**Answer:** We have now updated the abstract per request. We have formulated the main limitations to read as follows (row 50-51):

Limitations of this study include the observational design, reliance on dispensation data, and the inability to study specific drug regimens.

**Please present your conclusions in the past tense (e.g., “there was a modest negative association between statin treatment and COVID-19 mortality”).**

**Answer:** We have now updated the conclusion to be past tense (row 53).

**Please state summary demographic information in the abstract and include relevant p-values.**

**Answer:** We have updated the abstract to include summary demographic information and relevant P-values for our main and sensitivity analysis (row 47 & 49).

**Please provide an ‘Author Summary’ section as outlined here: <https://journals.plos.org/plosmedicine/s/revising-your-manuscript>**

**Answer:** We have now provided an author summary which reads as follows:

**Why Was This Study Done?**

- Lipid-lowering HMG-CoA reductase inhibitors (statins) are commonly used and important for medical prevention. Their use during the pandemic has been queried since statins may influence COVID-19 mortality due to their pleiotropic effects on coagulation and the immune system.
- Previous studies of statins and COVID-19 mortality have, however, been inconsistent, so we aimed to clarify this relationship using total population data with detailed information and complete follow-up.

**What Did the Researchers Do and Find?**

- A cohort of 963 876 residents of Stockholm, Sweden, were followed from the 1^st^ of March 2020 until the 11^th^ of November 2020 using Swedish health registers.
- Prescription dispensation data were matched to health care data and the Swedish Cause of Death Registry, and analysed using multivariable cause-specific survival analysis.
- Statin treatment was associated with a moderately lower risk of COVID-19 mortality (HR, 0.88; 95% CI, 0.79-0.97), after accounting for a series of pre-existing health conditions and other factors. The association was corroborated by sensitivity analyses and did not vary importantly across risk groups.

**What Do These Findings Mean?**

- These findings suggest that statin treatment may have a modest preventive therapeutic effect on COVID-19 mortality, although randomized trials are needed to determine the causality of the observed association.
- In total, the findings support the continued use of statins for conditions such as cardiovascular disease and hyperlipidaemia, in line with current recommendations, during the COVID-19 pandemic.

**Please remove the ‘Conflicts of interest’ and ‘Role of the funding source’ from the title page of the manuscript. If published, the relevant information will be included as metadata based on your responses to the submission forms.**

**Answer:** We have now removed the COI and funding statement from the title page.

**Please update lines 191-192 to read “However, when adjusting for confounders, statin treatment was associated with a moderately lower risk of COVID-19 mortality (HR, 0.88; 95% CI, 0.79-0.97)”. Please similarly adjust other relevant statements throughout the manuscript (e.g. lines 221-222).**

**Answer:** We have updated the lines 222-223 and 256-257 to read as suggested. We have also updated the second paragraph of the discussion to read in a similar matter (row 261-268).

Furthermore, we realize that we have synonymously used “therapy” and “treatment” throughout the manuscript. Seeing as the editor seem to prefer “statin treatment” over “statin therapy”, we have now changed this throughout the manuscript.

**If a protocol and/or statistical analysis plan were prepared in advance of this study, please could these be provided as supplementary files with the next revision. Please refer to any attached protocol document in the Methods section (main text) and highlight any analyses that were not prespecified.**

**Answer:** We appreciate this comment seeing as we promote the use of protocols and statistical analysis plans in observational research – and we believe that PLOS has contributed to this positive development. However, we did not prepare a protocol in advance of our study given the priority to rapidly execute our study during the ongoing pandemic. Seeing as we support the shift towards preregistration in observational research we have included a sentence at the end of the statistical analysis section which reads as follows (row 191-192):

The rapid development of the COVID-19 pandemic prevented us from preregistering our statistical analysis plan, as we prioritized a rapid execution of our study.

**Please make sure references do not contain any bold/italicized text and use “et al.” after listing the first six authors of a paper.**

**Answer:** We have now changed our referencing style to Vancouver, ensured that the references do not contain any bold/italicized text, and included the use of “et al.” after listing the first six authors of a publication (row 351-515).

**In the reference list, please add “[preprint]” to all preprints, e.g., reference 26.**

**Answer:** We have now included “[preprint]” after the title of each preprint reference (row 418-420).

**Thank you for providing a completed STROBE checklist. In the checklist, please refer to individual items by section (e.g., "Methods") and paragraph number, not by line or page numbers as these generally change in the event of publication.**

**Answer:** We have now updated the included STROBE checklist per editor suggestions.

Reviewer #1:

**This is a well-executed observational study investigating the hypothesis that statins improve D-related mortality. The extent of the study as well as the rigorous approach are wellcomed.**

**Answer:** We appreciate the reviewer’s endorsement of our study and the acknowledgement of our effort to perform a rigorous evaluation of statins role in COVID-19 mortality.

**Some of the limitations are well-explained. For example, the type of statin used was not explored, which is a missed opportunity seen the differences between the statines. Moreover, it is not explained why this sub-analysis was not done.**

**Answer:** We concur with the reviewer that our inability to study different types of statins is a limitation, as we also acknowledge in the original manuscript (row 281-282). The reason for not performing such analysis is lack of power, and the inherent limitations that come from performing analysis of small samples.

We have now clarified that this is the reason for not performing such analysis, modifying our original section on dosage, type, and branding to read as follows (row 322-325):

Lastly, we have not been able to study possible effects of dosage, type or brand of statins. Specifically, despite our study being the hereby largest study of statins and COVID-19 mortality, we had an insufficient sample size to perform such granular analysis. As such, our estimate should be interpreted as the mean of possibly heterogeneous effects.

**Also, the exposure was defined as one or more collected prescriptions: if only one prescription? Also, information about the continuation till "outcome" seems missing: what if in the last days-weeks, no statines were anymore given?**

**Answer:** We appreciate the reviewer’s comment on individual prescriptions, and compliance until “last days-weeks”, as we believe that these issues are central to our methodological reasoning and rigour.

In our study, we have classified individuals as exposed based on their first collected prescription during a one year period, 1 March 2019 and 29 February 2020, immediately preceding the pandemic, regardless of whether they continue to collect prescriptions. As such, it is indeed possible for an individual to be classified as exposed having only collected one prescription. Including individuals at any given point if they collect a prescription, regardless of whether they collect subsequent prescriptions, is per an intention to treat (ITT) principle. This form of analysis is in accordance with the best practice of only including a) initiators of drug use that were b) intended to treat. We adopted this strategy to ensure the methodological rigour of our study.

An alternative analysis to the ITT analysis would be a per-protocol analysis. In a per-protocol, we would ensure that only individuals who are compliant with treatment/allocation are studied. However, this would lead to a selection of individuals, as non-compliant individuals would be excluded. Typically, as in the emulated target trial literature (see for example Dickerman et al., reference 28 in revised manuscript), this selection bias is addressed by inverse-probability weights (IPW). Using IPW one can, in theory, use prognostic factors of non-compliance to recover an unbiased estimate of the causal effect under a per-protocol analysis. However, this relies on the key assumption that prognostic factors of non-compliance can be accurately captured, an assumption which we do not believe is realistic (especially when utilizing electronic health records). Therefore, we conducted no per-protocol analysis.

We should note, as we assume that the reviewer is alluding to, that an ITT analysis with low compliance will lead to an attenuation of the causal effect estimate of exposure (as in the case where only one prescription is collected). As such, our analyses will represent a conservative estimate of the causal effect of statin use – while retaining the interpretation as the causal effect of an ITT analysis. We believe, as is also standard practice in clinical causal estimation, that the ITT represent the estimate most in line with the clinically most relevant question: what would be the expected effect if a clinician prescribed statins to a patient? This question, in itself, includes non-compliance to treatment strategies.

Nonetheless, as we see how others may share the questions by the reviewer, we have included a section expanding on the rationale for such analysis and its implications, and relating it to the special circumstances of the pandemic (row 287-298):

We also avoid confounding that may arise from altered behaviour during the COVID-19 pandemic. Specifically, the end of the period of exposure was set to 1 March 2020 – the start of the pandemic in Stockholm – since the collection of prescriptions during a pandemic could be a proxy for certain behaviours affecting the risk of exposure to the virus (e.g., the frequenting of public spaces). While we believe this is a major strength of our study, such time restrictions imply that we include statin dispensations which, in theory, could be distal from a COVID-19 infection. Indeed, individuals who collect a sole prescription in early 2019 will be classified as exposed. However, this is in line with the intention-to-treat principle. As such, our estimate may represent a conservative estimate of the causal effect of statins on COVID-19 mortality; our estimate can be interpreted as that obtained from an observational intention-to-treat analysis in the presence of non-compliance, which we believe is the clinically relevant causal contrast.

**Although one could ask these and other questions, I still think this is a valuable study worth to be published, adding to the "pleiotropic" effects of statines**.

**Answer:** We are grateful for the endorsement of the reviewer, and its important contribution to the clarity of our manuscript. We hope that our elaborations please the reviewer.

Finally, we appreciate that the reviewer highlights the pleiotropic effects of statins and concur that our study adds to the understanding of this class of drugs. Despite the biological relevance of statins for immunological and coagulative processes, few studies have been able to study their immediate influence on infectious diseases. Hence, our study has relevance for future research on the role of statins for other health states (including other infectious diseases and future pandemics).

Reviewer #2:

**The authors have done study statin in COVID-19. My comments are**

**1. Should be stated that chronic statin user before got COVID-19 affect the mortality.**

**Answer:** We concur with the reviewer that statin use is associated with a protective effect on all-cause mortality (as also demonstrated in the positive outcome analysis, row 474-278 in original manuscript). Furthermore, as we believe the reviewer is alluding to, this may render certain types of analyses susceptible to bias. Such bias, arising from the better survival of statin users and therefore a greater risk to be infected by COVID-19 is, generally, limited to cross-sectional analyses (i.e., logistic regression) and survival analysis which do not incorporate competing risks (i.e., the Kaplan-Meier method or the standard Cox regression). Here, we have employed a cause-specific Cox regression model. In such analysis, the time-at-risk is restricted to the outcome of interest (COVID-19 mortality), and all individuals are censored at relevant competing events (deaths from other causes).

The cause-specific Cox regression model is an appropriate model for aetiological inquiries and yields an approximate estimate of the causal effect in presence of competing events. This has been described in general epidemiology (Lau et al. and Andersen et al.), reiterated in clinical epidemiology (Latouche et al.), and of importance to our study, this has been recognized in vascular epidemiology (Austin et al.).

We would like to note that we received a detailed competing risk question from Reviewer #4. If the reviewer wishes to see an extended rebuttal on competing risks we refer to our reply to point 2 by Reviewer #4.

*References:*

Andersen PK, Geskus RB, de Witte T, Putter H. Competing risks in epidemiology: possibilities and pitfalls. Int J Epidemiol. 2012;41(3):861-870.

Austin PC, Lee DS, Fine JP. Introduction to the Analysis of Survival Data in the Presence of Competing Risks. Circulation. 2016;133(6):601-609.

Latouche A, Allignol A, Beyersmann J, Labopin M, Fine JP. A competing risks analysis should report results on all cause-specific hazards and cumulative incidence functions. J Clin Epidemiol. 2013;66(6):648-653.

Lau B, Cole SR, Gange SJ. Competing risk regression models for epidemiologic data. Am J Epidemiol. 2009;170(2):244-256.

**2. There was little data regarding how long did statin give protection. And this was retrospective cohort study, there was bias that could not be controlled. This issue should be added as the limitation of the study.**

**Answer:** We appreciate that the reviewer has raised this issue, as we believe that it may deserve further clarification in our manuscript. It is indeed true that we have not studied the extent to which statins specifically have short-term, intermediate, or long-term effects. Instead, we have quantified an aggregate measure of the preventive therapeutic effect. As described in the original manuscript (row 283-285), we did not investigate the possible effect of statins when (experimentally) used for the treatment of an ongoing COVID-19-infection. We, instead, set out to clarify any possible preventive effect of statin initiation in the year before the pandemic, using an intention to treat principle.

We wish to note that although our study is one of the studies with the longest follow-up times (most prior studies are restricted to early pandemic cases, see introduction row 82-87), neither we nor other investigators can study the long-term effects of statins as the duration of the pandemic have only lasted a little over a year. We share the reviewer’s opinion that the long-term effects and granular details of drug effects on COVID-19 should be targets for future investigations. Thus, we have now included a sentence that explicitly states this and encourages future investigations (row 326-330 in the revised manuscript):

This study does not concern the possible effect of statin therapy for treatment of severe COVID-19, but its possible preventive effect. Furthermore, although our duration of follow-up is longer than comparable studies, future studies could shed light on the long-term effects of statins on COVID-19 mortality and possible heterogeneity in effects over subsequent COVID-19 waves.

**3. Was there any that regarding the safety of statin users?**

**Answer:** We are thankful for the comment on the safety of statins, and recognize that the safety profile of statins has been (and should be, considering their use for medical prevention) of major debate throughout the past decade. We note that the topic has become less controversial in recent years due to scientific advances (as were cited in our original manuscript, row 289), and that these are reflected in statements by cardiovascular societies (e.g., American Heart Association, the European Society of Cardiology and the European Atherosclerosis Society).

We would like to underline, however, that the safety of statins is outside the scope of our current study. This is since the issue would require specifically designed and detailed analyses, dedicated to the different and many research questions embedded in the overall concept of "statin safety”.

However, we much agree with the reviewer that the safety of statins has relevance for the interpretation of our findings. We have thus supplemented our Discussion section as follows (row 331-338 in the revised manuscript):

Although we emulated a target trial with an intention-to-treat analysis, the study does not provide an analysis of the risk of adverse events in potential target groups. Safety evaluations are outside the scope of the current study as they require extensive clinical and qualitative safety data, and such evaluations should address several aspects of the overall concept of statin safety. Nonetheless, this has been addressed in previous clinical trials, but not in connection with this new potential indication (COVID-19 prevention). Although statins are considered to have a favourable safety profile [reference to Wood et al.] compared to many other drug classes discussed in connection with COVID-19, a clinical risk assessment must always be made.

*References:*

Wood FA, Howard JP, Finegold JA, et al. N-of-1 Trial of a Statin, Placebo, or No Treatment to Assess Side Effects. N Engl J Med. 2020;383(22):2182-2184.

**4. Different type of statin use may give different effect --> should also be discussed**

**Answer:** We agree with the reviewer that different types of statins could, in principle, give rise to different effects. Therefore, we acknowledge this as a limitation in our original manuscript and describe that the estimate of our study should be interpreted as the mean of possibly heterogeneous effects (row 281-282). Unfortunately, despite being the largest study to date, we did not have a sufficient sample size to perform such an analysis.

Following your comment, and a comment by Reviewer 1 (point 1), we have now expanded our original section on dosage, type, and branding to clarify why we did not perform any analyses of specific statins (row 322-325 in the revised manuscript):

Lastly, we have not been able to study possible effects of dosage, type or brand of statins. Specifically, despite our study being the hereby largest study of statins and COVID-19 mortality, we had an insufficient sample size to perform such granular analysis. As such, our estimate should be interpreted as the mean of possibly heterogeneous effects.

**5. In this study was interesting, dementia, obesity and diabetes was higher in statin group. This issue should be discussed DOI: 10.1016/j.amjms.2020.10.026**

**Answer:** Dementia, obesity, and diabetes were, more common in the statins group. This is expected from the indications for statin use (cardiovascular prevention among risk groups, including those with cardiometabolic syndromes, per se linked to these outcomes). These factors may indeed influence COVID-19 mortality, and thus constitute confounders in our analyses. Therefore, we chose to control for these conditions in our multivariable Cox regression (see original manuscript row 138-148). As such, our adjusted hazard ratio represents an estimate which is independent of these comorbidities, to the extent that they are captured by the health care system. This may not always be the case, and we are particularly concerned about the extent to which obesity is diagnosed. Hence, we dedicated a paragraph in the discussion section to that topic (see original manuscript row 266-272). Here, we also highlight that our study is observational, and as such may be subjected to residual confounding. Lastly, it can be argued that such residual confounding would lead to an underestimated benefit of statins and that our findings are conservative.

**6. The routine use of anticoagulant higher in statin group may give a confounder effect**

**Answer:** We concur with the reviewer that concurrent use of anticoagulant therapies may induce confounding, seeing as they are more prevalent among statin users in our study (5.0% vs. 2.1%). Therefore we chose to control for anticoagulation dispensations (ATC: B01A) (see original manuscript row 143 and appendix row 479). As such, the adjusted hazard ratio represents the effect where we control for the presence of anticoagulant concurrent use – removing its confounding effect.

**7. How about the use of diabetes drugs? some give benefit <https://doi.org/10.1007/s40200-021-00777-4>**

**Answer:** We are thankful for the reviewer bringing this important study to our attention. We concur that diabetes, and in principle diabetes drugs, could be relevant for COVID-19 pathology. As such, we chose to control for both type I and type II diabetes (see row 145 in the original manuscript).

However, the meta-analysis that the reviewer refers to demonstrate that Dipeptidyl Peptidase-4 (DPP-4) inhibitors are not associated with “composite poor outcomes of COVID-19”. Therefore, it is unlikely that DDP-4 inhibitors are a confounder of the relationship between statins and COVID-19 mortality, seeing as they have no association with the outcome. Yet, it could be possible that other diabetes drugs (such as metformin) have some association with the outcome. However, these will be – to an almost perfect extent – collinear with diabetes status. Thus, the additional control for such compounds will not change our estimates. Put in other words, the confounding of such compounds is captured by the ancestral confounding of diabetes status – rendering any further control for diabetes drugs inappropriate and potentially a source of increased bias.

Nonetheless, we share the Reviewer’s general concern that there is existing confounding in the association between statin use and COVID-19 mortality (as raised in this and other comments). Precisely therefore we have attempted to control for a vast number of confounders (row 138-148 in the original manuscript). Despite such efforts, we acknowledge in the original manuscript that our study, as an observational study, is always subject to residual confounding and that our findings should be interpreted with caution (row 266-272 in the original manuscript).

**8. In table 1 should give p value to know if both group was same or differ in characteristics**

**Answer:** The Strengthening the Reporting of Observational Studies in Epidemiology (STROBE) guidelines explicitly state that inferential measures should not be reported with descriptive characteristics:

Inferential measures such as standard errors and confidence intervals should not be used to describe the variability of characteristics, and significance tests should be avoided in descriptive tables. Also, P values are not an appropriate criterion for selecting which confounders to adjust for in analysis; even small differences in a confounder that has a strong effect on the outcome can be important*.* - in Vandenbroucke et al. (2007)

To the best of our knowledge, PLOS Medicine adheres to the STROBE guidelines.

If it pleases the reviewer and editors, we can include P-values of descriptive characteristics throughout our manuscript. However, we do not find them informative, reporting them does not align with reporting standards, and they tend to be overinterpreted by readers. We note that almost all P-values of such descriptive tests are less than 0.01, seeing as we have great statistical power. This does not reflect “true” differences - as the P-values of such descriptive tests are confounded by all other non-shared characteristics.

*References:*

Vandenbroucke, J. P., E. von Elm, D. G. Altman, P. C. Gøtzsche, C. D. Mulrow, S. J. Pocock, C. Poole, J. J. Schlesselman, M. Egger and S. I. for the (2007). "Strengthening the Reporting of Observational Studies in Epidemiology (STROBE): Explanation and Elaboration." PLOS Medicine 4(10): e297.

Reviewer #3:

**The authors present a large, Stockholm, Sweden population-based observational study of statin use and COVID-19 mortality. The study population included all residents of Stockholm over 45 yrs of age without liver disease. The exposure was any collection of a statin prescription from March 2019 through Feb 2020; outcome was death from COVID-19. They found that although unadjusted analysis revealed higher mortality for statin users (a sicker population), after extensive adjustment for confounders, statin use was associated with a reduction in COVID-19 mortality. Sensitivity analyses revealed this association to hold true for non-COVID deaths; for COVID-19 as the main cause of death; and after restricting analyses to statin users who initiated their statin use in the year prior to COVID.**

**I commend the authors for this interesting study. The large sample size allows for a thorough adjustment for covariates/potential confounders.**

**Answer:** We are humbled by the reviewer’s endorsement of our study and we wish to extend our gratitude for the reviewer’s thought-provoking comments which has greatly improved the clarity of our manuscript.

**Comments:**

**1. Abstract - HR is listed as 0.88 - the authors need to specify that this is the adjusted HR.**

**Answer:** We are thankful for the reviewer bringing this to our attention and we have now clarified that this is the adjusted hazard ratio (row 47 & row 49).

**2. Fig 1 should present the coefficients from the model which adjusts for confounders. The main analysis should be presented in the main figure. Similarity across age groups, sexes, risk groups, etc. should be tested using interaction terms in the main model. The current Fig 1 can be used as a sensitivity analysis**

**Answer:** We are happy to have received this opportunity to clarify what our Fig 1 depicts.

Firstly, we would like to clarify to the reviewer that we present both unadjusted and confounder adjusted hazard ratios for the sake of completeness, and as we believe that the divergence between unadjusted and adjusted analyses is in itself interesting (also highlighted as interesting by Reviewer 4).

Secondly, Figure 1 only contains estimates of the effect of statins on COVID-19 mortality. As such, the “overall” estimate is the main analysis of the effect of statins on COVID-19 mortality in the whole cohort (as also referred to in text, see row 189-192 in the original manuscript). Furthermore, in Figure 1 we present a series of subgroups estimates (as described in row 192-194 in the original manuscript), still estimating the effect of statins of COVID-19 mortality – but now within each stratum. For example, the estimate in the subgroup 80+ (adjusted HR 0.84) represents is the contrast between statin users and non-users among those who are older than 80. In other words, the unadjusted and confounder adjusted hazard ratio of statin use among those older than 80. We perform such analyses as complementary analyses as these factors have been prognostic of COVID-19 mortality, as they may affect the effect heterogeneity of statins, and as they speak to our generalizability.

To clarify that Figure 1 presents both crude and adjusted estimates of associations between statins and COVID-19 mortality, from the main as well as the stratified analyses, we have now edited the heading of Figure 1 to read as follows (row 239-240):

Hazard ratios of death from COVID-19 in relation to dispensations of statins overall, and the hazard ratios of death from COVID-19 in relation to dispensations of statins within strata of age, sex and within COVID-19 risk groups.

We appreciate that the reviewer wishes that we employ an interaction term (so-called cross-product term) for each stratum to quantify possible differences (“similarity”) across strata. Such an analytical strategy will yield a similar conclusion to that employed. However, we believe that such an approach is not the most optimal analytical strategy to address our clinical question. To be specific, we have a clinical concern that there exists general effect heterogeneity over the analyzed COVID-19 risk groups (and other strata), but we have limited interest in the divergence of any specific groups in comparison to another. As such, we believe that a homogeneity test is more suitable, such as the employed Cochran’s Q test, as it is more uniform to our concern of general heterogeneity. If we were to assess heterogeneity using cross-product terms, per reviewer suggestion, there would be *a priori* reason to be concerned that small divergences in substrata’s would result in findings that could be interpreted as “true” effect heterogeneity. Post hoc, however, as we identified no heterogeneity, we know that we would have reached the same conclusion using interaction terms. Furthermore, we see some communicative benefit with performing joint homogeneity tests compared to repetitive p-values for cross-product terms, which may lead readers to be sceptical of the number of tests performed. Finally, we acknowledge that there may be some efficiency benefits to the product term approach. We feel, however, that the benefits of the Cochran’s Q approach outweigh such efficiency concerns – especially in our large sample setting.

**3. The use of the term 'emulated target trial' is very unclear. Either explain this thoroughly, with references, in the methods, or drop the term. Please explain simply what this analysis is and what it shows.**

**Answer:** We are thankful that the reviewer has pointed out that we have not been thorough in our description of the emulated target trial framework, and that we have not referred to seminal emulated trial papers. Simply put, framing investigations with a hypothetical target trial in mind can reduce self-inflicted injuries that often plague epidemiology. Below we expand on the emulated target-trial framework and exert a revised section of our manuscript.

In brief, the emulated target trial framework is a recently developed approach (spearheaded by Miguel Hernán and others – see reference below) that has gained widespread recognition and momentum. The approach has proven incredibly useful when reasoning about various selection mechanisms when attempting to estimate causal effects in the observational setting. Specifically, the approach outlines that one should attempt to ‘emulate’ a hypothetical target trial using observational data. By doing so, researchers are less likely to induce self-inflicted bias – bias such as immortal time bias, depletion of susceptibles and mediator control, which has typically plagued observational studies. Generally, it has been shown that observational studies can recover the same estimates as randomized controlled trials in many scenarios, despite still being vulnerable to confounding.

In our study, we have designed the totality of our investigation to emulate a target trial of the primary prevention effect of statin on COVID-19 mortality. Specifically, and in contrast to prior studies of statins and COVID-19, we have spent considerable effort to ensure that we do not give rise to such self-inflicted injuries. For example, we do not induce immortal time bias by defining exposure on some future event (such as continuous dispensation of statins), we ensure that we do not condition on possible mediators (such as health status at ICU admission), and avoid conditioning on future events such as positive-PCR test. In the original manuscript, we alluded to these as issues in prior studies in the introduction (row 80-81), and further expanded on them in the discussion (row 241-242 & 246-248).

One of the emphasized aspects in the emulated target trial framework is the study of drug initiators, rather than including patients already using a therapy. The reasoning is as follows: we should attempt to emulate trials and in clinical trials we do not include patients who are already receiving the intervention of interest. When including so-called ‘prevalent users’ (those already using a therapy) we may induce bias by depletion of susceptibles. Specifically, prevalent users constitute a patient population who have survived on therapy long enough to be included in the study – which may give rise to selection bias. In our main analyses, we diverge from the emulated target trial framework in that we include prevalent users (making us susceptible to such bias). We chose to do this as the inclusion of prevalent users greatly increases the eligible population (increasing sample size). Nonetheless, seeing as this may induce bias, we restrict our cohort down to statin initiators in sensitivity analysis – thereby following the principles outlined in the emulated target trial framework. Therefore, we refer to our sensitivity analysis as an “emulated target trial” while retaining classical epidemiological terminology in the main manuscript.

We have now inserted a citation to a seminal paper describing the framework in the methods section (row 197) and accompanying appendix (row 520). Furthermore, to clarify what an emulated target trial is, and to accommodate the reviewer, we have extended the description of the emulated target trial in the appendix, a section which now reads as follows (row 518-530):

We base the design of our investigation on the so-called emulated target trial framework - a framework utilized to minimize self-inflicted injuries when estimating causal effects using observational data (reference to Hernan & Robins 2016, see below). Specifically, we attempt to emulate (replicate) a hypothetical trial of the primary prevention effect of statin on COVID-19 mortality. However, in contrast to a hypothetical trial, we chose to include prevalent statin users. This greatly enhances the eligible population but may lead to the inclusion of a selected population of therapy sustainers (and depletion of susceptibles).

To alleviate such bias, we repeat our analysis excluding prevalent users. Specifically, we emulated a target trial by excluding all individuals who received one or more prescriptions for statins during the year before the start of the study period (1 March 2018 – 28 February 2019). This means that only new users (initiators), not prevalent users, were included in the analysis. The analysis did not take into account possible discontinuation of treatment during the study period, meaning that the emulated trial was done according to the intention-to-treat principle.

*References:*

Hernan MA, Robins JM. Using Big Data to Emulate a Target Trial When a Randomized Trial Is Not Available. *Am J Epidemiol*. 2016;183(8):758-764.

**4. Positive control analysis- the authors state that"**

**"Our positive outcome analysis (benchmark) was consistent with a protective effect of statins (as expected), although the estimated protective effect was greater than that of the benchmark."**

**This statement is unclear- Please change to something like:**

**"In a similar model, the estimated protective effect of statins for non-COVID deaths was greater than for COVID deaths. "**

**Please state this plainly. Please present the model, in the appendix.**

**These results needs to be highlighted in the abstract and discussion. It suggests confounding, in that statin users are generally healthier than non-statin users, and this explains the observed protective effect in COVID.**

**Answer:** We are thankful for this comment, especially as we believe that our positive control analysis is an important contribution and that its use is novel in COVID-19 studies. However, we do not share the concern of the reviewer. We think that this concern may be a misinterpretation of our analyses, on behalf of our less clear justification in the appendix (row 463-473 in the original manuscript). In short, we use the “positive outcome” analysis as a tool to control the validity of our findings: If we can simply reproduce a “positive”, as in a known (that could be protective as well as a harmful effect), association that has been established from meta-analyses of large scale RCTs, we are more reassured that we handle bias and other pitfalls that plague observational studies of prescribed drug use satisfactorily.

The sentence that the reviewer suggests compares non-COVID deaths and COVID deaths. This is not an appropriate qualitative comparison based on our positive outcome analysis. The estimate obtained from the positive outcome analysis (HR, 0.77; 95% CI, 0.73-0.82) should be compared to that of the meta-analysis of randomized controlled trials of statins as primary prevention (RR, 0.91; 95% CI, 0.83-1.01). As such, we find that the association between statins and non-COVID mortality is similar to that of meta-analysis of randomized controlled trials (although marginally more protective). As the findings are in line with what we would expect (we are able to reproduce an established association), we are reassured that there is limited bias in our positive outcome analysis. Thus, we are more inclined to believe that our COVID-19 mortality analysis does not suffer from differential error (e.g., from confounding).

However, to clarify the sentence that the reviewer refers to, we have rephrased our text in the result section to read as follows (row 246-251):

Our positive outcome analysis was consistent with a protective effect of statins (as expected), although the estimated protective effect was greater than that of meta-analysis of randomized controlled trials examining statins for primary prevention in high-risk populations. The fact that we were able to replicate the known association between statin treatment and reduced (overall) mortality gives further support for the validity of the results from our COVID-19 analysis.

Furthermore, we have removed the term “benchmark” throughout the manuscript as this term is not well-defined and its removal enhances clarity (row 167, 215-216, 462-463 & 478 in the original manuscript).

During this revision, we have become aware that we did not refer to any methodological literature supporting our analysis. As such, we have now included a reference to Tchetgen Tchetgen who provides a comprehensive introduction to control outcome calibration (the larger framework that includes positive outcome analysis) (row 199, 549 & 558). We note that the definition provided by Tchetgen Tchetgen is in line with our original description (row 467-473):

A positive control outcome can be defined for an outcome with a well-established nonnull causal association with the exposure, which is confounded in the observed sample by a subset of unobserved confounders for the exposure effects on the primary outcome. Positive control outcomes can […] be used to detect unobserved confounding by verifying whether the known association is replicated in the observed sample. - Tchetgen Tchetgen

Finally, we are not sure what the reviewer means by “present the model, in the appendix”. If the reviewer refers to presenting the findings of our analysis in the appendix we already do so (see row 474-478 in the original manuscript). We do not wish to include the totality of coefficients, as we see no justification for such presentations, and as this leads readers to erroneous interpretations and the so-called Table 2 fallacy – an issue which has plagued medical literature (see for example Westreich and Greenland).

In summary, we do not share the perception that our positive outcome analysis indicates confounding, despite it being marginally more protective than the meta-analysis of RCTs. In fact, the positive outcome analysis makes us more inclined to believe that our conservative conclusion is correct. Nonetheless, we agree with the reviewer that our original description lacked clarity, which we have now addressed.

*References:*

Tchetgen Tchetgen E. The control outcome calibration approach for causal inference with unobserved confounding. Am J Epidemiol. 2014;179(5):633-640.

Westreich D, Greenland S. The table 2 fallacy: presenting and interpreting confounder and modifier coefficients. Am J Epidemiol. 2013;177(4):292-298.

**5. There is significant potential for misclassification here - What if only 1 or 2 statin prescriptions were obtained, in early 2019 - this would not be likely to influence outcomes. Why start so early on? I would recommend that the criteria be that a prescription was picked up in the 3 months prior to COVID; or at the very least, perform a sensitivity analysis for those with at least 1 obtained from ~Jan 2020 on? The authors state this could cause additional biases, however the small risk of that does not seem to outweigh the large likelihood of the misclassification with the current methodology.**

**Answer:** While we judge the temporal restriction of exposure a strength of our study, we appreciate the reviewers concern that may indeed be shared by other readers. Hence, we are grateful for the opportunity to make changes to manuscript for clarification.

First, our protocol adheres to the recommended strategy of analyzing observational data according to the principles of an experimental, randomized, study. Thus, while an individual who only collected one prescription in 2019 is unlikely to have a long-term benefit on Covid-19 mortality, him or her is truly “exposed” (and not misclassified) as per our protocol, designed to produce an unbiased estimate of the intention-to-treat effect in the presence of non-compliance. We argue that this is the appropriate causal target.

Were we, however, to study the effect of concurrent filling of statin prescriptions during the pandemic we expect a bias to occur (e.g., defined by 3 months prior to COVID, per reviewer suggestion), and such bias to lead to a departure of estimates of associations from the null (either towards infinity or towards zero). We do not share the reviewer’s position that such bias has a “small risk”, rather, we argue that this bias would be unrecoverable and of large magnitude. Similarly, we believe that one of the challenges in interpreting previous studies in the field is that most studies have studied exposure during the pandemic (studies of statins and other drugs). Therefore, as we have also stated in the original manuscript (row 253-261), we believe that our ability to avoid this bias is an important strength of our investigation.

The origin of this bias becomes apparent when considering the extreme scenario that we are in. Specifically, the pandemic constitutes a scenario where normal behaviours cannot be expected. Notably, the spread of SARS-CoV-2 is different by communities, individuals are affected differently by non-pharmaceutical interventions, and groups in society have substantially different resilience to cope with the ongoing pandemic. This complex and extreme scenario can be expected to substantially influence health-seeking behaviour and there is no reason to believe that this is uniformly distributed over confounders of statins and COVID-19 mortality (e.g., socioeconomic status). This is a form of temporal confounding that will be unrecoverable – we are therefore very sceptical of such analysis.

The reviewer also suggests an analysis where we restrict to those who collect a prescription from Jan 2020 until March 2020, thereby ensuring temporal proximity to possible infection. We agree that this is a sound analysis since it will not be affected by the bias process described above. We note, however, that an analytical strategy based on statin use being defined as dispensations during the three months preceding the pandemic, including also prevalent users would be necessary to meet requirements of statistical power (e.g., not excluding those with prior statin prescription), would probably produce findings very similar to those presented in our study. This is as statins are commonly prescribed for long-term (or even life-long) use once initiated according to current guidelines. Finally, the ideal analysis might be to study statin use initiated during the suggested period only. We appreciate the reviewer’s suggestion to conduct this analysis, and it was what we aimed to achieve when we designed our original initiation analyses (the target trial emulation) (row 440-445 in the original manuscript). Unfortunately, despite the large sample size and long follow-up (comparing to other studies on Covid-19 mortality), numbers did not allow for this approach why we extended the enrolment period to gain sufficient statistical power.

In summary, we chose to favour a conservative estimate of the causal effect (towards null) rather than allowing bias to drift our estimate upwards or downwards. We appreciate that this concern, and our conservative strategy, may not be immediately clear to all readers. Therefore, we have chosen to elaborate on our analytical strategy and underscore that our estimate may be conservative. We have now, to accommodate the reviewer’s and others’ comments, included a section in the discussion which reads as follows (row 287-298):

We also avoid confounding that may arise from altered behaviour during the COVID-19 pandemic. Specifically, the end of the period of exposure was set to 1 March 2020 – the start of the pandemic in Stockholm – since the collection of prescriptions during a pandemic could be a proxy for certain behaviours affecting the risk of exposure to the virus (e.g., the frequenting of public spaces). While we believe this is a major strength of our study, such time restrictions imply that we include statin dispensations which, in theory, could be distal from a COVID-19 infection. Indeed, individuals who collect a sole prescription in early 2019 will be classified as exposed. However, this is in line with the intention-to-treat principle. As such, our estimate may represent a conservative estimate of the causal effect of statins on COVID-19 mortality; our estimate can be interpreted as that obtained from an observational intention-to-treat analysis in the presence of non-compliance, which we believe is the clinically relevant causal contrast.

Reviewer #4:

**This is an interesting study on the assoication between statin therapy and COVID-19 mortality using the population data from Stockholm. Although well written, there are a few major issues needing attention.**

**Answer:** We would like to thank the reviewer for his or her comments, thereby giving us the opportunity to clarify and improve the readability of our manuscript. We further appreciate that the reviewer found our manuscript well written - despite needing some clarifications.

**1) The key findings are in Figure 1. We can see the effect of statin changed the direction from increasing the risk (OR 2.02) to protective (OR 0.88) after adjustment of all the confounders. The effect of 0.88 with a p-value of 0.01 is marginal and close to borderline significance. While this still might be possible after adjustment, we really have to scrutinise the statistical analyses to make sure we leave nothing to chance. Also in Figure 1, after adjustment, we can see that over 80 yrs old, IHD, hypertension and heart failure all have protective effect (reduce the risk) of Covid death. Why? This is quite difficult to comprehend and interpret.**

**Answer:** We are thankful for this opportunity to comment on our key findings. The reviewer raises several important points, including the magnitude of effect, the change in estimates between crude and adjusted, and the specific estimates within certain strata. Below we shall outline a reply to each of these important concerns.

First, we would like to point out that we agree with the reviewer that our findings are indicative of a “marginally” protective effect. However, we think that this finding is more important than the reviewer give credit to. Specifically, we know from an abundance of literature that statins are greatly beneficial for improving cardiovascular health. If statins have some marginal benefit for reducing COVID-19 mortality, it would be welcomed – but their benefit for vascular health remains (as reflected by our conclusion). However, and perhaps more critically, if statins had caused an increased risk for COVID-19 mortality it would be detrimental – seeing as there is a great number of statins users in the general population, and current guidelines may need immediate revision. In the introduction, we highlight the extremities in estimates from previous studies (e.g., row 85-87 in the original manuscript, OR 6.21 vs OR 0.46), which demonstrate that – prior to our study – there are reasons to be concerned. However, as we find a *marginally* beneficial effect, and perhaps more importantly no *substantial excess risk*, we conclude that our findings “… supports the continued use of statin therapy for medical prevention according to current recommendations also during the COVID-19 pandemic.” (row 55-57 in the original manuscript). We believe that this is an accurate representation of our findings and that our study provides a rigorous evaluation of Statins in COVID-19, and that our findings are reassuring for those concerned that statins are associated with an excess risk of COVID-19 mortality.

Secondly, the reviewer raises the fact that there is a discrepancy (and reversal of direction) between the crude and adjusted analysis (i.e., 2.02 vs 0.88). We do not share the view by the reviewer that this warrants caution (or more “scrutiny”). To be precise, we would expect this reversal given what we know about the confounding structure of this population. Specifically, as elegantly put by Reviewer 3, statin users are merely “a sicker population”. When not controlling for imbalances in covariates (crude analysis), we would be perplexed if we did not observe excessive risks of COVID-19 mortality among the statins users. Especially seeing as they, by their indication for statins, should suffer from cardiovascular morbidity – a known determinant of COVID-19 severity. As such, contrary to the reviewer’s position, we are reassured when we observe an attenuation of the excessive risk, as this is precisely what we would expect to observe in the presence of positive confounding.

Finally, the reviewer has suggested that some of the findings in Figure 1 are difficult to comprehend and interpret. Although we believe that we have been clear in the result text (row 189-194 in the original manuscript) and when summarizing our findings in the discussion (row 221-226 in the original manuscript), we believe that lack of clarity in the Figure 1 heading may have given rise to a misunderstanding of the estimates (also reflected in one comment by Reviewer 3). Specifically, the estimates in Figure 1 are the within-strata effect of statins on COVID-19 mortality. That is, we are comparing users and non-users of statins among those, for example, over 80 years old – where we find that statins are associated with a moderate benefit (adjusted HR 0.84). We believe that the reviewer erroneously, because of our lack of clarity, interpret these estimates as the effect of being over 80 years old – which we agree would be surprising if we found to be beneficial for COVID-19 mortality.

To clarify that Figure 1 presents the main analysis, and stratified analysis, of the effect of statins on COVID-19 mortality we have clarified the Figure 1 heading to read as follows (row 239-240):

Hazard ratios of death from COVID-19 in relation to dispensations of statins overall, and the hazard ratios of death from COVID-19 in relation to dispensations of statins within strata of age, sex and within COVID-19 risk groups.

**2) Competing risk. As the outcome of survival analysis (Cox model) is Covid death rather than all-cause mortality, the competing risk from other deaths needs to be addressed in the analysis. Therefore, survival analysis taking into account of competing risk should be performed as main analysis. As the current result is close to borderline significance and also changes the direction of effect after adjustment, the competing risk issue becomes even more important to make sure the results are robust and reliable.**

**Answer:** We are thankful for this important comment, and for allowing us to clarify our estimator.

We would like to clarify that we employ a cause-specific Cox regression. Such a model is not invariant of competing events, since it censors them. This is an established approach for the estimation of causal effects in the presence of competing events, which we consider appropriate as argued below. As such, our main analysis is a “survival analysis taking into account [the] competing risk[s]” – in line with the reviewer’s recommendation. Although we initially described that we end follow-up at deaths (row 125-126 in the original manuscript), we believe that our phrasing may lend itself to misinterpretation. As such, we have now clarified that we follow “until death from COVID-19, other deaths, or 11 November 2020, whichever occurred first” (row 154-155 in the revised manuscript) and we have rephrased our statistical section to ensure compatibility with the literature (rephrasing “Cox regression” to “cause-specific Cox regression”, row 165 in the revised manuscript).

For the sake of completeness, however, we feel the need to expand on alternative methods for competing risk which we believe that the reviewer may be alluding to. Specifically, we assume that the reviewer may be referring to other competing risk models typically utilized in the literature, such as estimators of the sub distributional hazard (Fine-Gray method).

While these methods may be important for some investigations (e.g., predictive inquiries), they have limited utility in causal inference (aetiology). This has been described in general epidemiology (Lau et al. and Andersen et al.), reiterated in clinical epidemiology (Latouche et al.), and of importance to our study, this has been recognized in vascular epidemiology (Austin et al.). Generally, the critique of such competing-risk models is that the underlying risk set does not lend itself to causal interpretations (Austin et al. and Andersen et al.). Specifically, the cause-specific Cox regression (which we employ) describes the:

…. instantaneous rate of occurrence of the *k*th event in subjects who are currently event free (ie, in subjects who have not yet experienced any of the different types of events) – Austin et al.

That is the effect among those who are still at risk of developing the event of interest. While the alternative models (Fine-Gray method) describe the

… instantaneous risk of failure from the *k*th event in subjects who have not yet experienced an event of type *k* – Austin et al.

That is the effect among those who have not experienced a certain event. The major distinction comes from the latter including individuals in the risk set who, in our example, would have died from other causes when estimating the effect on COVID-19 mortality. Clearly, the cause-specific model which only includes individuals who are *totally* event-free (competing events and events of interest) in the risk set is an appropriate estimator of the causal effect.

In conclusion, although hazard estimators have their challenges (as all models), we firmly believe that the cause-specific estimator is ‘more’ appropriate than alternative models of the hazard (i.e., the Fine-Gray method) – a position that is supported by methodological and applied literature.

*References:*

Andersen PK, Geskus RB, de Witte T, Putter H. Competing risks in epidemiology: possibilities and pitfalls. Int J Epidemiol. 2012;41(3):861-870.

Austin PC, Lee DS, Fine JP. Introduction to the Analysis of Survival Data in the Presence of Competing Risks. Circulation. 2016;133(6):601-609.

Latouche A, Allignol A, Beyersmann J, Labopin M, Fine JP. A competing risks analysis should report results on all cause-specific hazards and cumulative incidence functions. J Clin Epidemiol. 2013;66(6):648-653.

Lau B, Cole SR, Gange SJ. Competing risk regression models for epidemiologic data. Am J Epidemiol. 2009;170(2):244-256.

**3) For adjusted confounders, why use age of 45 as a cut-off for inclusion?**

**Answer:** We are thankful for the reviewer’s comment, as we realize that we lacked some clarity when explaining why we chose to only include individuals older than 45. This was predominantly done for two reasons:

1. To effectively exclude all pregnant women (99%), seeing as statin use is contraindicated in pregnancy.
2. To reduce the number of non-informative index persons. Specifically, although young individuals constitute a large part of the population of Stockholm, at the time of the study there were very few COVID-19 deaths under the age of 45 (n ≈ 10). In designing our study, we were concerned that the inclusion of these individuals could lead to instability in covariate control (partly due to dispersion and partly due to violations of positivity).

Although we initially described that the low age limit effectively excluded pregnant women (original manuscript row 110-114), we have now clarified that this was one of the specific reasons for imposing such limits. We have further clarified that excluding young individuals who, at the time of the study, had a close to zero baseline risk of COVID-19 mortality enhances stability in covariate control. The section now reads as follows (row 136-143 in the revised manuscript):

The study included all individuals aged 45 or older, residing in Stockholm county in 2019 as well as 1 March 2020, as recorded in the Total Population Registry. We chose to only study those older than 45 to effectively exclude > 99 per cent of pregnant women, as statins are contraindicated during pregnancy. Furthermore, the lower age limit enhances the stability of our covariate control, as there had been few COVID-19 deaths among young people during the study period. Furthermore, statins are contraindicated in active liver disease, individuals diagnosed with liver disease (ICD 10: K70-K77) up to 5 years prior to the start of the study (i.e. 1 Jan 2015 or later) were hence excluded (n = 2 103).

**Is there ethnicity information available?**

**Answer:** We appreciate the reviewer’s comment, and realize that ethnic subgroups have been particularly affected by the pandemic. Registration of ethnicity is, however, prohibited by law in Sweden. Thus, this information was not available to us. Instead, we adjust our analyses for other sociodemographic factors such as country of birth, education, area of residence, and disposable income. These factors are captured in detail from registers kept by Statistics Sweden, and do, to some extent serve as proxies for ethnicity and indeed for other socially stratified health conditions, lifestyle and behaviours that have shown to be strong risk factors for COVID-19 in Stockholm (as per our regional reporting in Swedish <https://www.folkhalsoguiden.se/globalassets/verksamheter/forskning-och-utveckling/centrum-for-epidemiologi-och-samhallsmedicin/folkhalsoguiden/rapporter-och-faktablad/rapport-2021.6-covid-19-i-stockholms-lan-till-och-med-mars-2021-tga-ef.pdf>) and across the world.

**4) In results in the abstract and also in the main text, it says "A total of 2 545 individuals died from COVID-19 during follow-up, including 765 (0.5%) statin users and 1780 (0.2%) non-users". However, where are these 0.5% and 0.2% coming from? I can't reproduce these percentages using any figures in Table 1. Can authors please clarity this?**

**Answer:** We appreciate that the reviewer brought this to our attention. The percentages represent the percent of COVID-19 deaths in each group. That is, for statin users:

$$\frac{765}{169642}100 \sim0.5$$

And for non-users:

$$\frac{1780}{794234}100 \sim0.2$$

To clarify this, we have rephrased the sentences to read as follows in the abstract (row 45-46):

A total of 2 545 individuals died from COVID-19 during follow-up, including 765 (0.5%) of the statin users and 1780 (0.2%) of the non-users.

And to read as follows in the result section (row 220-221):

A total of 2 545 individuals died from COVID-19 during follow-up, including 765 (0.5%) of the statin users and 1,780 (0.2%) of the non-users (unadjusted HR, 2.02; 95% CI, 1.85-2.20) (Figure 1).

**5) In Table 1, for age, median and IQR should be presented in the form of xx (yy-zz) instead.**

**Answer:** We are thankful that the reviewer brought this to our attention. The median and IQR for age in Table 1 now reads as follows:

| **Characteristic** | **Statin users**  **(N=169 642)** | **Non-users**  **(N=794 234)** |
| --- | --- | --- |
| **Age** |  |  |
| Years, Median (IQR) | 71.0 (64-78) | 58.0 (49-67) |
