# Supplementary material for: HMG-CoA reductase inhibitors and COVID-19 mortality in Stockholm, Sweden: A registry-based cohort study
Source: PLoS Med. 2021 Oct 14;18(10):e1003820. doi: 10.1371/journal.pmed.1003820 (PMC8516243; doi:10.1371/journal.pmed.1003820)
Supplement: Attachment — Submitted filename: V2 Rebuttal - Point by Point.docx [file pmed.1003820.s006.docx]

Requests from the editors:

**Please update the language of your Author Summary as follows:**

**•	Bullet point 1: Lipid-lowering HMG-CoA reductase inhibitors (statins) may influence COVID-19 mortality via their pleiotropic effects on coagulation and the immune system.**

**•	Bullet point 2: Previous studies on this topic have been inconsistent, so we performed a population-based cohort study to investigate the relationship between statins and COVID-19 mortality.**

**•	Bullet point 3: Using data from Swedish health registers, a cohort of 963876 residents of Stockholm, Sweden, were followed from the 1st of March 2020 until the 11th of November 2020.**

**Answer:** We have now updated the first three bullet points to the editor suggested phrasing.

**Please consider changing the word ‘importantly’ in bullet point 5 of your Author Summary (line 79) to ‘substantially’ or similar. Alternatively, you could say ‘The association was corroborated by sensitivity analyses and was relatively consistent across risk groups’.**

**Answer:** We have now changed the bullet point to read as follows (page 4 row 71 in the revised manuscript):

The association was corroborated by sensitivity analyses and did not vary substantially across risk groups.

**For the final bullet point of your Author Summary (line 84), please replace the word ‘total’ with ‘conclusion’ or ‘summary’.**

**Answer:** We have now changed to “summary” instead of “total”.

**Line 117: Please change ‘set in the rich Swedish health registers’ to ‘using Swedish health register data’, or similar.**

**Answer:** We have now changed the relevant section to read as follows (page 6 row 114-117 in the revised manuscript):

… we present a large population-based observational study using Swedish health register data, with the aim of determining the relationship between statin treatment and COVID-19 mortality, …

**Line 117: Please change ‘to determine’ to ‘of determining’.**

**Answer:** We have now changed the relevant phrasing.

**Line 139: Remove the erroneous hyphen in the word ‘pregnant’.**

**Answer:** We have now removed the erroneous hyphen.

**Line 142: Remove the erroneous hyphen in the word ‘contraindicated’.**

**Answer:** We have now removed the erroneous hyphen.

**Citations should be in square brackets, and preceding punctuation. Please update throughout.**

**Answer:** We have now updated the citations accordingly.

**Comments from the reviewers:**

**Reviewer #4: Many thanks authors for their effort to improve the manuscript. Most of my comments were satisfactorily addressed by the authors. However, there is still one remaining issue - the competing risk issue. I am not at all convinced/satisfied with the authors' response on competing risk. To simply censor the other deaths is inadequate and will not solve the competing risk problem in the cause-specific Cox models. To address the competing risk in the Covid death survival analysis, models such as Fine-Gray method are needed as there are potentially quite a few non-Covid deaths in the cohorts which present as competing risk. While it's appreciated that authors gave some references for the arguement, but the vast majority of the stats literature supports the use of the Fine-Gray method (for example) for cause specific survival analysis to effectively and adequately address the competing risk issue.**

**Answer:** We are happy that the reviewer feels satisfactorily addressed by a majority of our rebuttal. Furthermore, we appreciate the opportunity to extend our prior discussion on competing risk models.

While we concur with the reviewer that much statistical literature has focused on Fine-Gray methods, to which we have also contributed, we still do not share the position that the Fine-Gray method (aka. The Subdistribution hazard model) is appropriate for causal inference. Nonetheless, as we realize that we differ in this position, and as we realize that other readers may share similar concerns as the reviewer, we have now performed the Fine-Gray method accounting for competing risks from other causes of death.

There was no major difference when utilizing the Fine-Gray method; most differences were beyond the second decimal of the hazard ratio associated with statin use (see Table below for exhaustive estimates). Specifically, we originally reported an overall adjusted effect of 0.88 (95% CI: 0.79-0.97) and we now found the Fine-Gray method to yield 0.88 (0.80-0.98) when adjusting for the same covariates. For the reviewer's interest, we also analysed non-Covid mortality using the Fine-Gray method (see Table below), where we find that statins have a protective effect - as would be expected.

As such, we conclude that accounting for competing risks through the Fine-Gray method did not alter our findings. We believe that the reason for this minimal difference stems from the fact that we do not have a very long follow-up period (as compared to studies of cancer, for example), that the effect of statins is likely to be in the same direction for all-cause mortality and Covid-19 mortality, and that the competing risk concerns raised by the reviewer primarily arise when attempting to estimate the marginal survival.

As we acknowledge that other readers may share similar concerns as the reviewer we have now included the Fine-Gray method in the supplementary material and described its use under the sensitivity analyses section in the methods (page 9 row 198-199 in the revised manuscript):

… and 4) performed our main analysis using the Fine-Gray method for competing risks [Reference to Fine and Gray 1999], treating non-Covid deaths as competing events.

Finally, we detail that there were no discrepancies between our primary analysis and the Fine-Gray method in the results section under the sensitivity analyses heading (page 14 row 253-255 in the revised manuscript):

There was no difference between our main analysis and when treating non-Covid deaths as competing events using the Fine-Gray method (adjusted HR, 0.88; 95% CI, 0.80-0.98) (see eTable 2 in Appendix).

After the below table, we reply to the additional questions posed by the reviewer.

|  | Number | | Number | | Cause-specific Cox regression | | Subdistribution hazard models | | | |
| --- | --- | --- | --- | --- | --- | --- | --- | --- | --- | --- |
|  | Covid-19 deaths | | Other causes of deaths | | HR (95% CI)  **[Original findings for reviewer convenience]** | | HR (95% CI)  **[Fine-Gray method]** | | | |
|  |  | |  | |  | |  | | | |
|  | Statin users | Non-statin users | Statin users | Non-statin users | Covid-19 death | | Covid-19 death | | Other causes of death | |
|  |  |  |  |  | Unadjusted | Fully adjusted | Unadjusted | Fully adjusted | Unadjusted | Fully adjusted |
| ***Overall*** | 765 | 1 780 | 2 350 | 6 149 | 2.02 (1.85-2.20) | 0.88 (0.79-0.97) | 2.01 (1.85-2.19) | 0.88 (0.80-0.98) | 1.80 (1.71-1.88) | 0.78 (0.73-0.82) |
|  |  |  |  |  |  |  |  |  |  |  |
| **Age** |  |  |  |  |  |  |  |  |  |  |
| *45-69* | 85 | 215 | 297 | 1 175 | 3.18 (2.48-4.09) | 0.79 (0.56-1.11) | 3.18 (2.48-4.09) | 0.79 (0.55-1.13) | 2.03 (1.79-2.31) | 0.63 (0.53-0.75) |
| *70-79* | 230 | 340 | 785 | 1 391 | 1.34 (1.14-1.59) | 0.89 (0.72-1.88) | 1.34 (1.14-1.59) | 0.90 (0.73-1.11) | 1.12 (1.03-1.22) | 0.71 (0.63-0.79) |
| *80+* | 450 | 1 225 | 1 268 | 3 583 | 0.71 (0.63-0.79) | 0.84 (0.74-0.95) | 0.71 (0.64-0.79) | 0.84 (0.74-0.96) | 0.68 (0.64-0,72) | 0.80 (0.74-0.87) |
| **Sex** |  |  |  |  |  |  |  |  |  |  |
| *Male* | 502 | 861 | 1 428 | 2 747 | 2.26 (2.03-2.53) | 0.89 (0.78-1.02) | 2.26 (2.02-2.52) | 0.90 (0.79-1.03) | 2.02 (1.89-2.15) | 0.75 (0.69-0.81) |
| *Female* | 263 | 919 | 922 | 3 402 | 1.65 (1.44-1.89) | 0.85 (0.73-1) | 1.65 (1.43-1.89) | 0.86 (0.73-1.00) | 1.56 (1.45-1.68) | 0.81 (0.74-0.88) |
| **Covid-19 risk group** |  |  |  |  |  |  |  |  |  |  |
| *No known risk factor* | 404 | 1 384 | 1 256 | 4 936 | 1.80 (1.61-2.01) | 0.84 (0.71-0.99) | 1.79 (1.61-2.00) | 0.90 (0.80-1.02) | 1.57 (1.47-1.67) | 0.80 (0.75-0.86) |
| *Ischemic Heart Disease* | 307 | 261 | 908 | 737 | 0.37 (0.31-0.44) | 0.75 (0.61-0.90) | 0.37 (0.32-0.44) | 0.75 (0.61-0.91) | 0.39 (0.35-0.43) | 0.67 (0.60-0.76) |
| *Hypertension* | 652 | 1 127 | 1 983 | 3 602 | 0.90 (0.82-0.99) | 0.84 (0.75-0.94) | 0.90 (0.82-0.99) | 0.85 (0.75-0.95) | 0.86 (0.81-0.90) | 0.75 (0.70-0.80) |
| *Diabetes type II* | 369 | 336 | 919 | 934 | 0.75 (0.64-0.87) | 0.86 (0.73-1.02) | 0.75 (0.65-0.87) | 0.86 (0.73-1.03) | 0.67 (0.61-0.73) | 0.69 (0.62-0.77) |
| *Chr lower resp tract dis* | 131 | 229 | 470 | 888 | 1.11 (0.90-1.38) | 0.86 (0.66-1.11) | 1.11 (0.90-1.38) | 0.86 (0.66-1.12) | 1.03 (0.92-1.15) | 0.80 (0.70-0.92) |
| *Stroke and TIA* | 31 | 70 | 79 | 256 | 2.10 (1.38-3.21) | 1.19 (0.7-2.03) | 2.10 (1.38-3.20) | 1.22 (0.70-2.12) | 1.47 (1.14-1.89) | 0.60 (0.44-0.83) |
| *Chr kidney failure st 3-5* | 9 | 44 | 41 | 114 | 0.97 (0.47-1.99) | 0.54 (0.21-1.4) | 0.97 (0.47-1.99) | 0.59 (0.25-1.40) | 1.71 (1.20-2.44) | 0.62 (0.38-1.02) |
| *Heart failure* | 236 | 412 | 793 | 1 367 | 0.60 (0.51-0.70) | 0.78 (0.64-0.94) | 0.60 (0.51-0.71) | 0.78 (0.64-0.95) | 0.60 (0.55-0.66) | 0.74 (0.66-0.82) |

**By the way, what is the all-cause mortality in the cohorts? and what's the number of other deaths? what's the proporiton of Covid deaths vs other deaths?**

**Answer:** There was a total of 3 115 deaths (1.8%) among the statin users and a total of 7 929 (1%) deaths among the non-users (see Table below). As we reported in the supplementary material in the original manuscript, there were 2 350 deaths from non-Covid causes among the statin users and 6 149 among the non-users.

|  | **Statin users**  **(N=169 642)** | **Non-users**  **(N=794 234)** |
| --- | --- | --- |
| Covid-19 deaths, No. (%) | 765 (0.5) | 1 780 (0.2) |
| Other causes of death, No. (%) | 2 350 (1.4) | 6 149 (0.8) |
| Total deaths, No. (%) | 3 115 (1.8) | 7 929 (1.0) |

**So far this competing risk issue hasn't been appropriately addrressed in the paper which could lead to the results and conclusions being subject to scrutiny.**

**Answer:** We hope that the reviewer finds the second rebuttal satisfactory and that the reviewer feels reassured that our conclusion is not subject to scrutiny due to competing risks. Finally, we would like to extend our gratitude to the reviewer for their thought-provoking comments and for enabling us to further reflect upon possible biases with our analyses.
