# Supplementary material for: HMG-CoA reductase inhibitors and COVID-19 mortality in Stockholm, Sweden: A registry-based cohort study
Source: PLoS Med. 2021 Oct 14;18(10):e1003820. doi: 10.1371/journal.pmed.1003820 (PMC8516243; doi:10.1371/journal.pmed.1003820)
Supplement: Attachment — Submitted filename: Rebuttal - Point by Point.docx [file pmed.1003820.s007.docx]

Requests from the editors:

**Please add the following statement, or similar, to the Methods: "This study is reported as per the Strengthening the Reporting of Observational Studies in Epidemiology (STROBE) guideline (S1 Checklist)."**

**Answer:** We have now included the relevant sentence in the Method section under the Study design and data sources heading (row 132-134).

**Line 206: Please include ‘years’ when stating median age of your sample**

**Answer:** The relevant section now reads as follows (row 208):

…median age of 71.0 years, compared to 58.0 years for non-users…

**Table 1: Please define the abbreviations IQR, TIA, and ACE in the footnotes**

**Answer:** We have now included a footnote of Table 1 reading as follows (row 219):

Abbreviations: ACE, angiotensin-converting enzyme; IQR, interquartile range; TIA, transient ischemic attack.

**Figure 1: TIA should be defined as transient ischemic attack (rather than ischemic attack, transient).**

**Answer:** We have now redefined TIA as transient ischaemic attack in the legend of Figure 1 (row 243).

**Lines 293, 295, 296, 514, 523, 527: Please remove the hyphens from words where they should not appear on these lines.**

**Answer:** We have now removed the relevant hyphens.

**Comments from the reviewers:**

**Reviewer #4: Many thanks authors for their great effort to improve the manuscript. The authors have addressed my concerns comprehensively. I am satisfied with the response and revision. No further issues needing attention.**

**Answer:** We are happy that the reviewer feels satisfactorily addressed by our rebuttal, recognizing our effort. We extend our gratitude to the reviewer for their allocated time, dedication to the review process and contribution to the quality of our manuscript.
